# Supplementary material for: Modelling the long-term fairness dynamics of data-driven targeted help on job seekers
Source: Sci Rep. 2023 Jan 31;13:1727. doi: 10.1038/s41598-023-28874-9 (PMC9889751; doi:10.1038/s41598-023-28874-9)
Supplement: Supplementary file 1 — Supplementary Information. [file 41598_2023_28874_MOESM1_ESM.pdf]

Appendix for “Long-term dynamics of fairness: understanding the impact of data-driven targeted help on job seekers”

A Intervention scenarios

Tab. A1: Overview of scenarios, defined by the k-matrix of the intervention model

Balanced, no targeting, no class dependent effect

	predicted high	predicted low
real high	1	1
real low	1	1

Only low, targeted solely on predicted low prosepct group, no class dependent effect

	predicted high	predicted low
real high	1	0
real low	1	0

Only high, targeted solely on predicted high prosepct group, no class dependent effect

	predicted high	predicted low
real high	0	1
real low	0	1

Balanced–errors-penalized, no targeting, effectivity only half if classification is incorrect

	predicted high	predicted low
real high	1	1/2
real low	1/2	1

B Overview of model parameters

Tab. B1: Parameters of the model

parameter	default value	description
N	10000	size of active population
α_{pr}	2	dependence of x_2 on x_{pr}
x_{max}	2	truncation value for data generation
ΔT_u	5	time that the lowprospect group is withdrawn from the active pool
T_u^{max}	100	time after which job-seekers that did not find a job leave the system
α_l	0	location of the logistic job-market function
β_l	10	steepness of the logistic job-market function
β_b	0 (unbiased), 2 (biased)	bias against the underprivileged group in the logistic job-market function
T_u^{γ}	10	threshold (in timeunites) that defines the border between highrospect and lowprospect group
x_1^{max}	2	maximum that skill-feature x_1 can reach in the interventio model
x_2^{max}	2	maximum that skill-feature x_2 can reach in the interventio model

C Model evolution plots

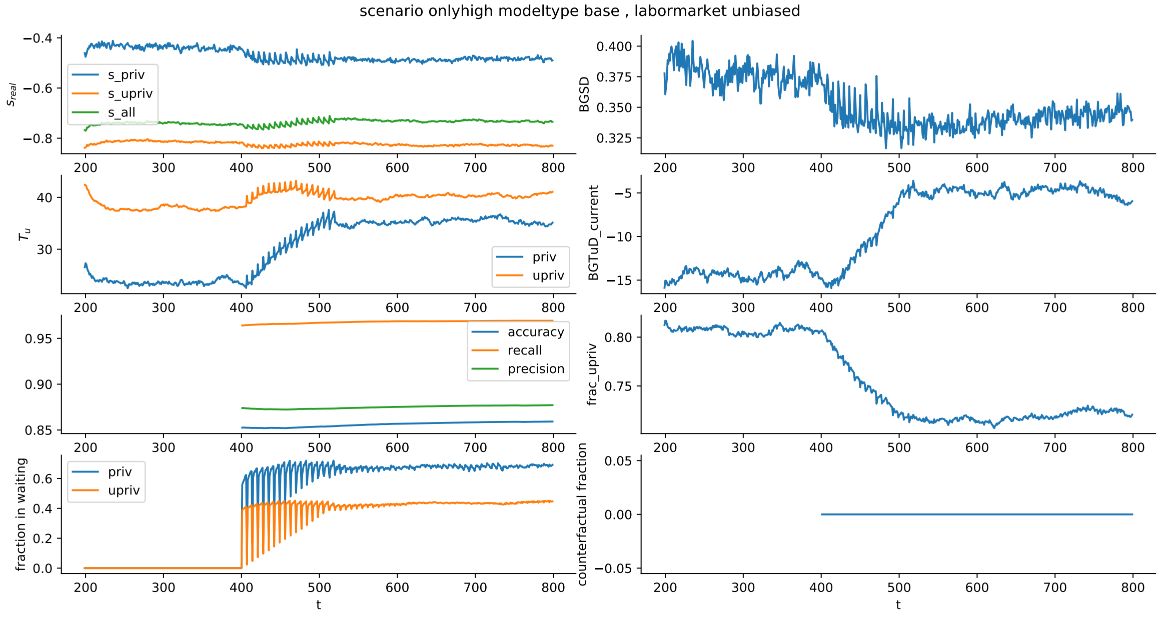

Fig. C1: time evolution of the base model with unbiased labor market and “onlyhigh” scenario.

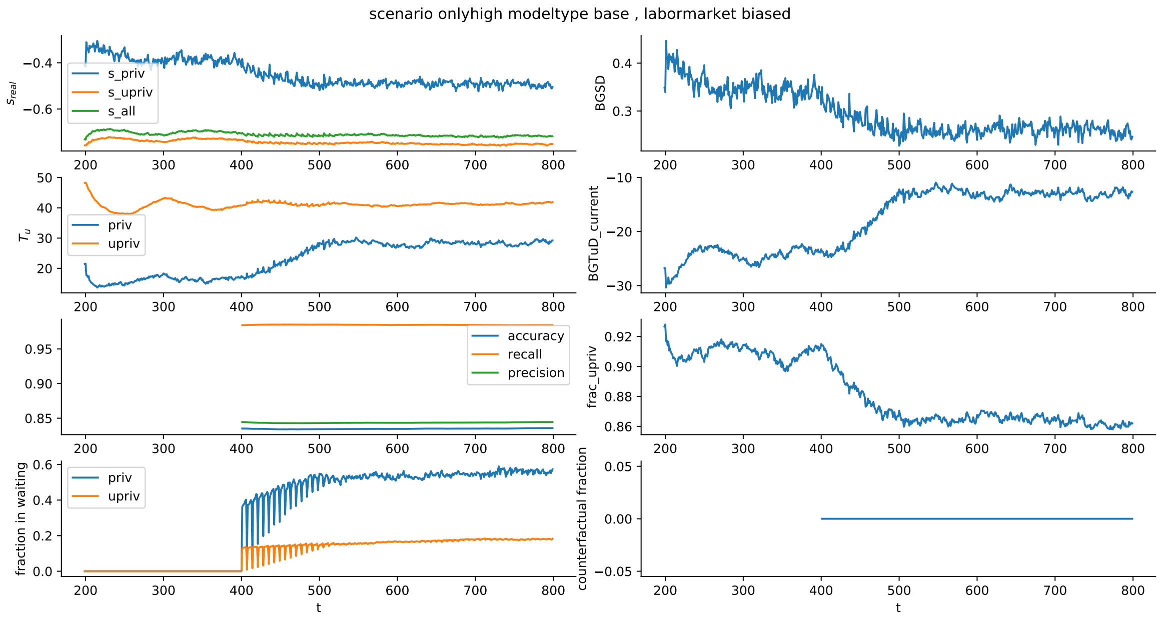

Fig. C2: time evolution of the base model with biased labor market and “onlyhigh” scenario.

D Runs with different parameters

Throughout the main paper, we used a fixed set of model parameters (except for the ones that were varied in the experiments, i.e. labor market bias and model type). Here we explore runs where (some) of the other parameters were changed as well.

D.1 ΔT_u

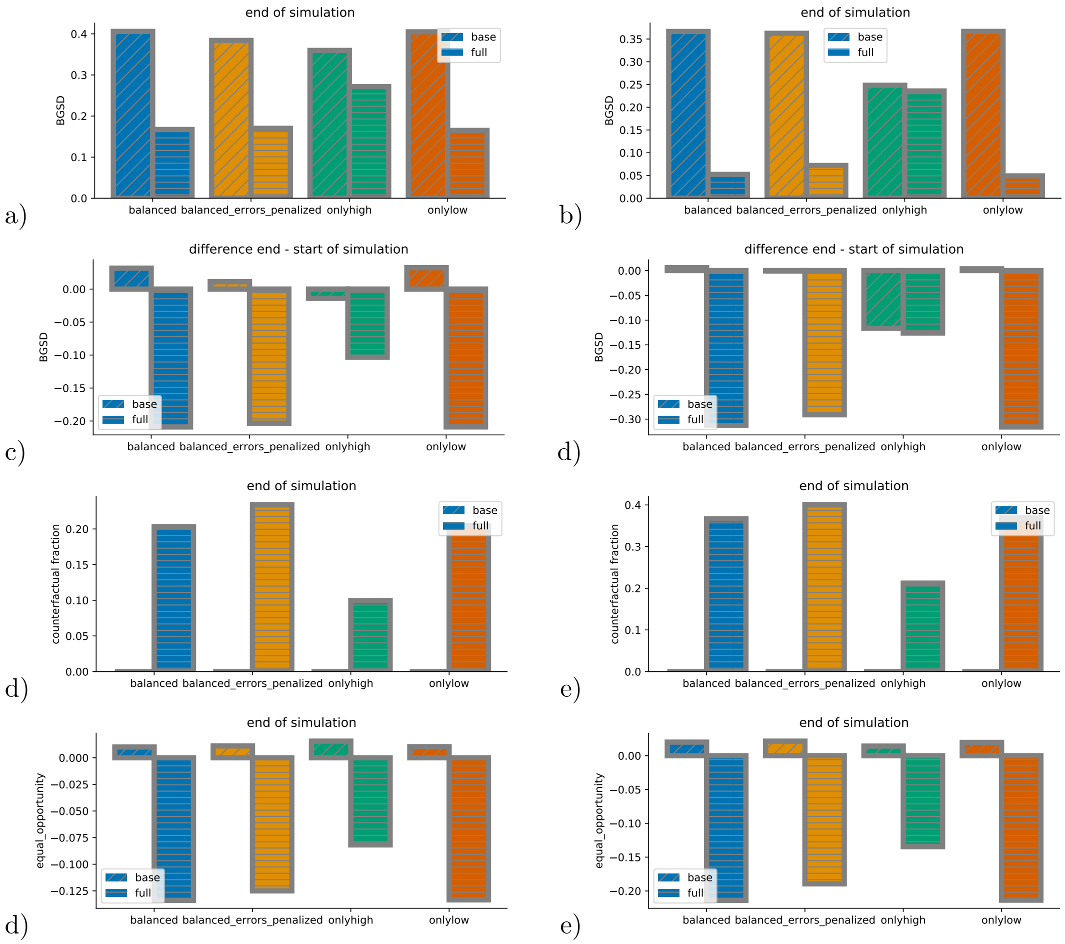

Fig. D1: Results for $\Delta T_u = 20$ ($\Delta T_u = 5$ in standard model configuration) *BGSD* at the end of the simulations (a,b) and change of *BGSD* from start to end of simulations (c,d), for the unbiased (a, c) and biased (b, d) labor market. Different colors indicate different intervention scenarios, and different hatching indicates the base (without protected attribute) and the full model (protected attribute)

D.2 Different T_u^γ

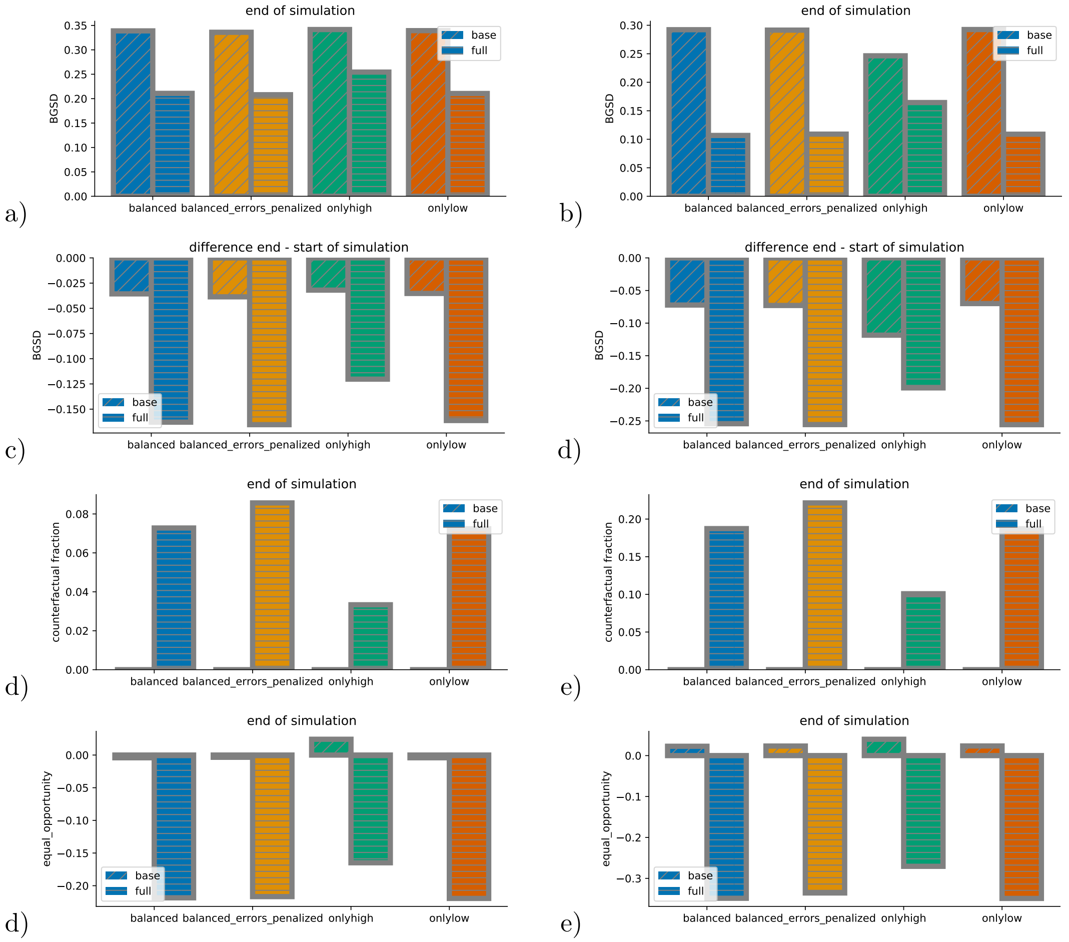

Fig. D2: Results for $T_u^\gamma = 15$ ($T_u^\gamma = 10$ in standard model configuration) *BGSD* at the end of the simulations (a,b) and change of *BGSD* from start to end of simulations (c,d), for the unbiased (a, c) and biased (b, d) labor market. Different colors indicate different intervention scenarios, and different hatching indicates the base (without protected attribute) and the full model (protected attribute)

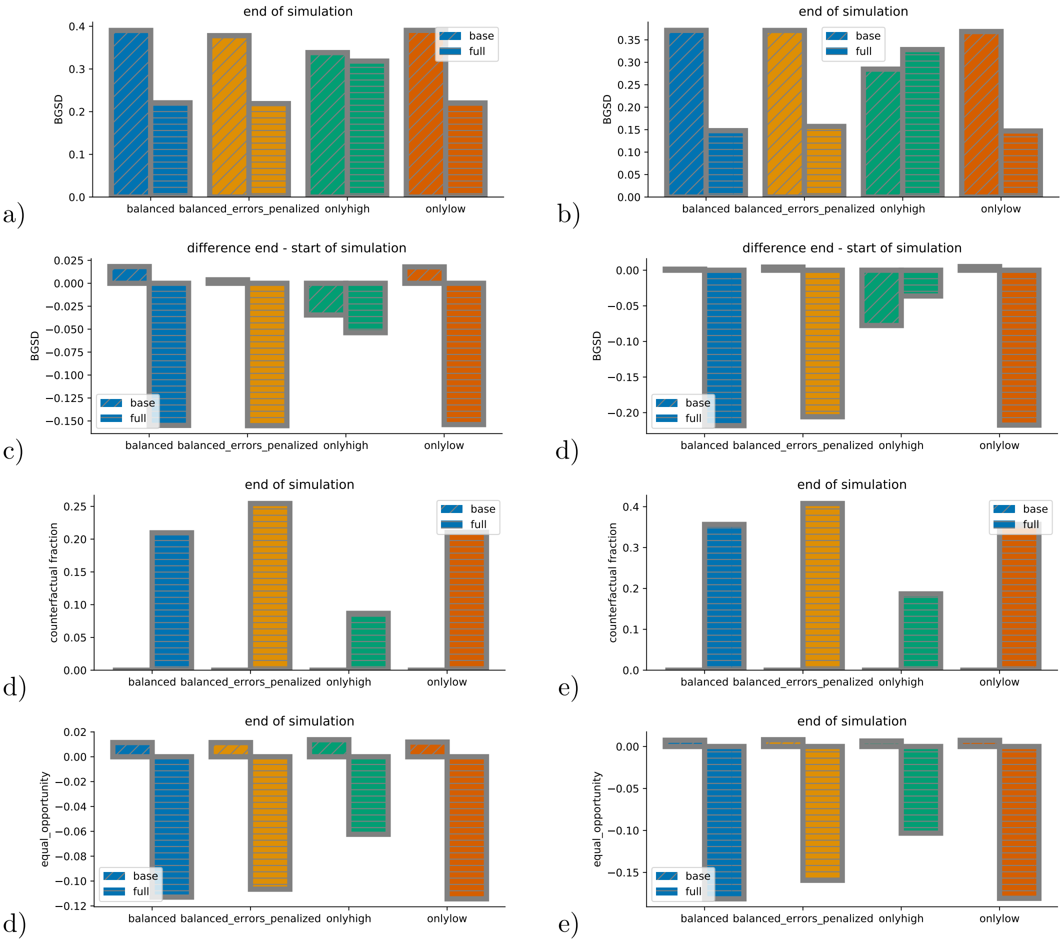

Fig. D3: Results for $T_u^\gamma = 15$ ($T_u^\gamma = 10$ in standard model configuration) *BGSD* at the end of the simulations (a,b) and change of *BGSD* from start to end of simulations (c,d), for the unbiased (a, c) and biased (b, d) labor market. Different colors indicate different intervention scenarios, and different hatching indicates the base (without protected attribute) and the full model (protected attribute)
